# Genistein Promotes Skeletal Muscle Regeneration by Regulating miR-221/222

**DOI:** 10.3390/ijms232113482

**Published:** 2022-11-03

**Authors:** Linyuan Shen, Tianci Liao, Jingyun Chen, Jianfeng Ma, Jinyong Wang, Lei Chen, Shunhua Zhang, Ye Zhao, Lili Niu, Changjun Zeng, Mailin Gan, Li Zhu

**Affiliations:** 1 Key Laboratory of Livestock and Poultry Multi-Omics, Ministry of Agriculture and Rural Affairs, College of Animal and Technology, Sichuan Agricultural University, Chengdu 611130, China; 2 Farm Animal Genetic Resource Exploration and Innovation Key Laboratory of Sichuan Province, Sichuan Agricultural University, Chengdu 611130, China; 3 Chongqing Academy of Animal Science, Chongqing 402460, China

**Keywords:** genistein, muscle regeneration, miR-222, miR-221

## Abstract

Genistein (GEN), a phytoestrogen, has been reported to regulate skeletal muscle endocrine factor expression and muscle fiber type switching, but its role in skeletal muscle regeneration is poorly understood. As a class of epigenetic regulators widely involved in skeletal muscle development, microRNAs (miRNAs) have the potential to treat skeletal muscle injury. In this study, we identified miR-221 and miR-222 and their target genes *MyoG* and *Tnnc1* as key regulators during skeletal muscle regeneration, and both were regulated by GEN. C2C12 myoblasts and C2C12 myotubes were then used to simulate the proliferation and differentiation of muscle satellite cells during skeletal muscle regeneration. The results showed that GEN could inhibit the proliferation of satellite cells and promote the differentiation of satellite cells by inhibiting the expression of miR-221/222. Subsequent in vitro and in vivo experiments showed that GEN improved skeletal muscle regeneration mainly by promoting satellite cell differentiation in the middle and late stages, by regulating miR-221/222 expression. These results suggest that miR-221/222 and their natural regulator GEN have potential applications in skeletal muscle regeneration.

## 1. Introduction

In daily life, skeletal muscles are often damaged by many injuries due to strenuous exercise and accidents, and need to be repaired to maintain their functions [1]. Skeletal muscle has a strong self-repair ability after injury, and this repair ability mainly depends on a myogenic precursor cell—the muscle satellite cell [2]. Muscle satellite cells exist between the muscle cell membrane and the basement membrane, and the number of satellite cells in uninjured skeletal muscle is small and in a quiescent state [3,4]. After skeletal muscle injury, under the action of various cytokines, muscle satellite cells are activated, migrate to the injury site, and then proliferate, differentiate, and fuse into new myotubes to complete the repair of skeletal muscle injury [5].

Skeletal muscle regeneration is a dynamic regulatory process. Skeletal muscle undergoes a series of complex processes from destruction, repair, to remodeling after injury, and a large number of cytokines are involved in this process [6]. The activation, proliferation, and differentiation of satellite cells are the most critical stages in the process of skeletal muscle regeneration. Quiescent satellite cells express *Pax7* and *CD34* [7]. After stimulation, quiescent satellite cells activate and enter the cell cycle, and generate activated satellite cells through asymmetric division [8]. Activated satellite cells express *MyoD*, rapidly proliferate under the action of cyclins and their regulators (cyclin-dependent kinases, CDKs), and then differentiate and fuse into myotubes under the control of myogenic regulatory factors [9]. In addition, macrophages also play an important role in skeletal muscle regeneration [10,11]. Macrophages are the main inflammatory cells in injured muscles, mainly by secreting inflammatory mediators such as *TNFα* and *IL6*, phagocytosing necrotic muscle tissue, and providing an attachment environment for new myotubes [12,13]. Overall, in the early stages of muscle injury, inflammatory cells and immune cells are recruited to the site of injury, M1 macrophages and T cells maintain a pro-inflammatory environment and clean up necrotic and damaged areas, and satellite cells are activated to enter the cell cycle [6,10]; in the middle and late stages of muscle injury, the pro-inflammatory environment is converted to an anti-inflammatory environment, dominated by M2 macrophages. M2 macrophages and regulatory T cells (tregs) promote the differentiation of satellite cells and maturation of newly formed muscle fibers until muscle damage repair is completed [8,11].

As important regulatory factors, miRNAs play an important role in biological processes such as skeletal muscle development, maintenance of muscle fiber morphology and structure, and muscle cell survival [14]. Studies have reported that miR-1, miR-133 and miR-206 are potential regulators in the regeneration process, and they are involved in skeletal muscle regeneration by affecting the activation, proliferation and differentiation of satellite cells [15,16]. These reports suggest that miRNAs are potential targets for regulating skeletal muscle regeneration, but limited by cost and technical bottlenecks, miRNAs are difficult to directly use in the treatment of skeletal muscle injury. Finding natural regulators that can specifically regulate the expression of miRNAs is a safe and effective strategy for the treatment of muscle injury.

Previous studies have reported that estrogen and its receptors play an important role in skeletal muscle regeneration [17]. Genistein (GEN) is a phytoestrogen that exerts estrogenic or antiestrogenic effects in mammals [18]. In previous studies, it was reported that GEN could play a role in inhibiting cancer cells [19,20], improving obesity [21], and modulating skeletal muscle development [22] by regulating the expression of miRNAs. In this study, we report that GEN can act as a natural regulator of miR-221 and miR-222. GEN promotes skeletal muscle regeneration in the middle and late stages after injury by regulating the expression of miR-221 and miR-222 and their target genes *MyoG* and *Tnnc1*. Our results provide a reference for the treatment of skeletal muscle injuries.

## 2. Results

### 2.1. GEN Treatment Promotes Skeletal Muscle Regeneration after Muscle Injury

**Figure 1:**
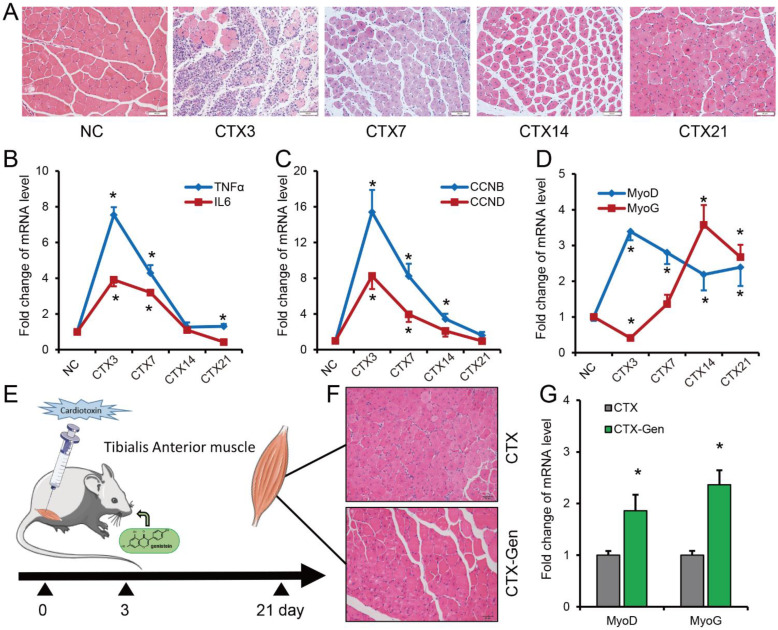
GEN promotes damaged-muscle repair. (**A**) Tibialis anterior (TA) muscle tissue sections of mice on day 0 (NC), day 3 (CTX3), day 7 (CTX7), day 14 (CTX14), and day 21 (CTX21) after cardiotoxin (CTX) injection. (**B**) The expression of *TNFα* and *IL6* in damaged muscle. (**C**) The expression of *CCNB* and *CCND* in damaged muscle. (**D**) The expression of *MyoD* and *MyoG* in damaged muscle. (**E**) A schematic showing skeletal muscle injury time, injury site, and GEN treatment time in mice. (**F**) Histological sections of TA muscle from GEN-treated (CTX-Gen) and no GEN-treated mice (CTX) 21 days after injury. (**G**) Expression of *MyoD* and *MyoG* in TA muscle of no GEN-treated and GEN-treated mice at 21 days of injury. *n* = 3. * *p* < 0.05. Scale ruler: 50 μm.

A mouse model of skeletal muscle injury was successfully constructed by injecting cardiotoxin (CTX) (Figure 1A). During the regeneration process of skeletal muscle injury, the expressions of *TNFα*, *IL6*, *cyclin B* (*CCNB)*, *cyclin D (CCND)* and *MyoD* all showed a trend of increasing first and then decreasing, while the expression of *MyoG* showed a trend of first decreasing, then increasing and finally decreasing (Figure 1B–D). We observed that 21 days after muscle injury, new muscle fibers filled the injury site (Figure 1A). In the GEN-treated group (CTX-Gen), the central nucleus myotubes began to decrease compared with the CTX group (Figure 1A,E–F). At the same time, the expression of *MyoD* and *MyoG* in the CTX-Gen group were significantly higher than that in the CTX group (Figure 1G). These results suggest that GEN treatment from day 3 after muscle injury can promote the regeneration process of skeletal muscle.

### 2.2. miR-222 and miR-221 Are Important Regulators of Skeletal Muscle Regeneration

**Figure 2:**
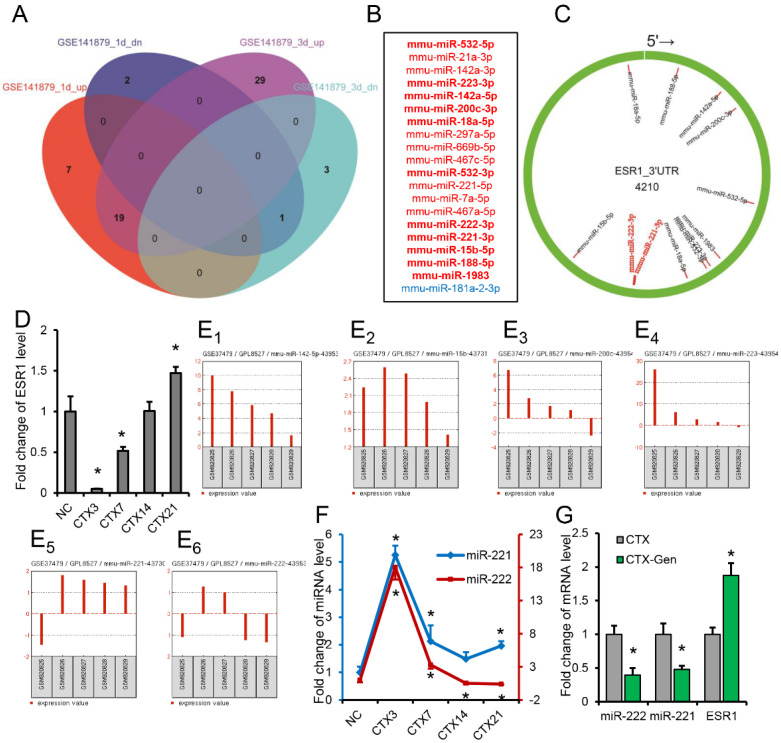
miR-221 and miR-222 are involved in skeletal muscle regeneration. (**A**) Venn diagram showing co-expression of miRNAs 1 day and 3 days after CTX injection (GSE141879). (**B**) Co-upregulated (red) and co-downregulated (blue) miRNAs. (**C**) Binding sites of co-upregulated miRNAs to the 3′UTR of *ESR1*. (**D**) Expression of *ESR1* during mouse skeletal muscle regeneration. (**E**) Expression of miR-142-5p (**E_1_**), miR-15b (**E_2_**), miR-200c (**E_3_**), miR-223 (**E_4_**), miR-221 (**E_5_**) and miR-222 (**E_6_**) during skeletal muscle regeneration in the GEO database (GSE37479, GSM920825 (day1), GSM920826 (day3), GSM920827 (day4), GSM920828 (day7), GSM920829 (day21)). (**F**) Expression of miR-221 and miR-222 during mouse skeletal muscle regeneration. (**G**) Expression of miR-221, miR-222 and *ESR1* in TA muscle of no GEN-treated and GEN-treated mice at 21 days of injury. (**D**,**F**,**G**) *n* = 3. * *p* < 0.05.

**Figure 3:**
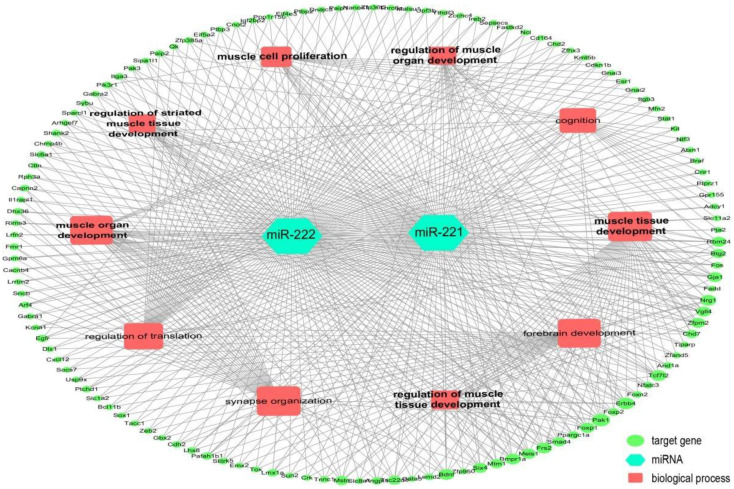
Regulatory networks reveal target genes of the miR-221/222 family and their involved biological processes.

By analyzing the GEO DataSets (GSE141879), it was found that 19 miRNAs were co-highly expressed at 1 day and 3 days after CTX injection, and 1 miRNA was co-down-regulated (Figure 2A,B). Interestingly, we found that 12 of the 19 up-regulated miRNAs could target *ESR1*. Meanwhile, the expression of *ESR1* was significantly down-regulated on day 3 after skeletal muscle injury (Figure 2D). The expression trends in these 12 miRNAs were further analyzed using the GEO database online analysis tool GEO2R (GSE37479). It was found that the expression of miR-142-5p, miR-15b, miR-200c, miR-223, miR-221 and miR-222 gradually decreased from 3 days to 14 days after CTX injection (Figure 2(E_1_–E_6_)), while the expression of *ESR1* gradually increased from 3 days to 14 days after muscle injury in this study (Figure 2D). Among them, miR-221 and miR-222 belong to the same gene family and have the same seed sequence, and *ESR1* has also been reported as their target gene [23]. Similar expression patterns of miR-221 and miR-222 were also shown in this study (Figure 2F). In addition, we also found that the expression of miR-221 and miR-222 was significantly decreased in the CTX-Gen group, while the expression of *ESR1* was significantly increased, compared with the no GEN-treated injury group (Figure 2G). Gene ontology (GO) analysis of the target genes of the miR-221/222 family showed that these target genes were mainly involved in the biological processes (BP) of muscle cell proliferation, muscle organ development, muscle tissue development, regulation of muscle organ development, regulation of muscle tissue development and regulation of striated muscle tissue development, which are directly related to skeletal muscle development (Figure 3). These results suggest that miR-221 and miR-222 have important roles during skeletal muscle development.

### 2.3. Effects of GEN and miR-221/222 Family on Myoblast Proliferation

**Figure 4:**
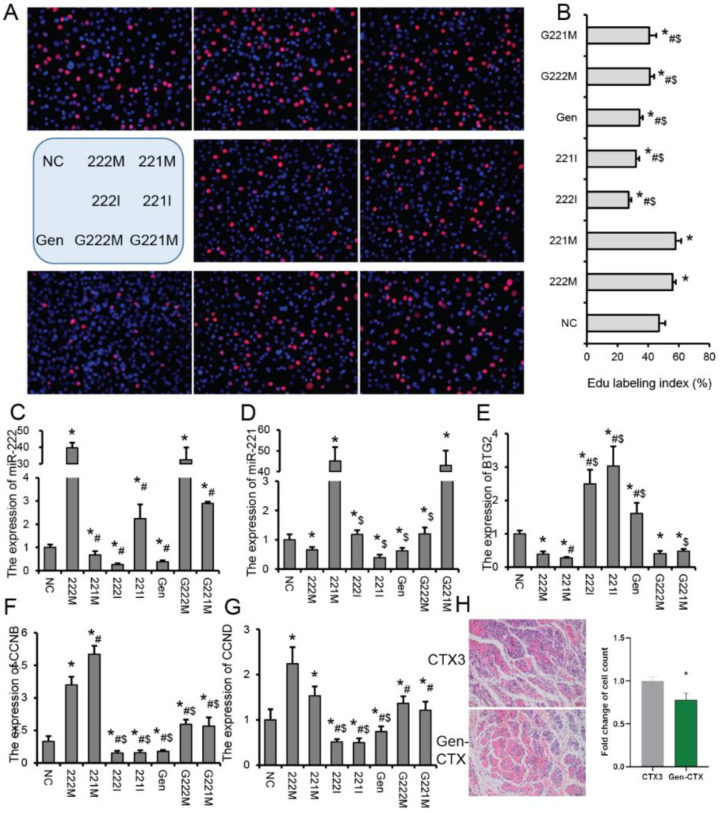
Effects of GEN and miR-221/222 family on myoblast proliferation. (**A**,**B**) EdU staining (**A**) and counting (**B**) of C2C12 myoblasts after different treatments. miR-221 mimic (221 M), miR-222 mimic (222 M), miR-221 inhibitor (221 I), miR-222 inhibitor (222 I), GEN (Gen), GEN + miR-221 mimic (G221 M) and GEN + miR-222 mimic (G222 M). (**C**–**G**) Expression of miR-222 (**C**), miR-221 (**D**), *BTG2* (**E**), *CCNB* (**F**) and *CCND* (**G**) in C2C12 myoblasts after different treatments. (**H**) Histological sections of TA muscle at 3 days after injury from GEN-treated (Gen-CTX, start treatment 3 days before muscle injury) and no GEN-treated mice (CTX). *n* = 3. * *p* < 0.05, compared with the NC group, # *p* < 0.05, compared with 222 M group, $ *p* < 0.05, compared with 221 M group.

Proliferation is an important stage of skeletal muscle regeneration. To further verify the effects of GEN and miR-221/222 family on C2C12 myoblasts, we transfected C2C12 myoblasts with miR-221 mimic (221 M), miR-222 mimic (222 M), miR-221 inhibitor (221 I), miR-222 inhibitor (222 I), GEN (Gen), GEN + miR-221 mimic (G221M) and GEN + miR-222 mimic (G222M), respectively. Compared with the NC group, EdU-labeled positive cells were significantly increased in the 221 M and 222 M groups (Figure 4A,B). Compared with the NC group, Gen, 221 I and 222 I groups had significantly fewer EdU-labeled positive cells (Figure 4A,B). Compared with the 221 M group, the EdU-labeled positive cells in the G221M group were significantly reduced (Figure 4A,B). Compared with the 222 M group, the EdU-labeled positive cells in the G222M group were significantly reduced (Figure 4A,B). These results indicated that miR-221/222 could promote myoblast proliferation, and GEN could inhibit the promoting effect of miR-221/222 on myoblast proliferation. We also observed that GEN treatment significantly inhibited the expression of miR-222 and miR-221 in myoblasts (Figure 4C,D). *BTG2* (BTG anti proliferation factor 2) is the target gene of miR-222 that we previously reported can inhibit cell proliferation [21]. In this study, we found that transfection of 222 M and 221 M significantly inhibited *BTG2* expression, while 222 I, 221 I and Gen significantly promoted *BTG2* expression (Figure 4E). Meanwhile, we found that 222 M and 221 M significantly promoted the expression of *CCNB* and *CCND* in C2C12 myoblasts, while 222 I, 221 I and Gen significantly inhibited the expression of *CCNB* and *CCND* (Figure 4F,G). Compared with 222 M and 221 M, the expression of *CCNB* and *CCND* was significantly inhibited by GEN treatment (Figure 4F,G). Correspondingly, we found that pretreatment of GEN before muscle injury reduced the number of cells at the muscle injury site in mice 3 days after injury (Figure 4H). Interestingly, we found that treatment of GEN prior to injury also resulted in reduced inflammatory infiltration at the injury site (Figure 4H). In RAW264.7 cells, the number of EdU-labeled positive cells was significantly increased in the 221 M and 222 M groups, compared with the NC group, and the EdU-labeled positive cells in the Gen group were significantly reduced (Supplementary Figure S1A,B). Meanwhile, GEN significantly inhibited the proliferation-promoting effects of 221 M and 222 M on RAW264.7 cells (Supplementary Figure S1A,B). Compared with the NC group, 221 M and 222 M significantly promoted the expression of *CCNB* and *CCND* in RAW264.7 cells, while Gen significantly inhibited the expression of *CCNB* and *CCND* (Supplementary Figure S1C,D). Meanwhile, GEN treatment significantly alleviated the promoting effects of 221 M and 222 M on the expression of *CCNB* and *CCND*, and the inhibitory effect on *BTG2* (Supplementary Figure S1C–E). In addition, we also found that Gen, 221 I and 222 I significantly inhibited lipopolysaccharide (LPS)-induced increases in expression of miR-221, miR-222, *TNFα* and *IL6* (Supplementary Figure S1F–J). These results suggest that GEN may affect the proliferation of RAW264.7 cells and LPS-induced inflammatory responses by regulating the expression of miR-221 and miR-222.

### 2.4. GEN and miR-221/222 Family Regulate Myoblast Differentiation and Fusion In Vitro and In Vivo

**Figure 5:**
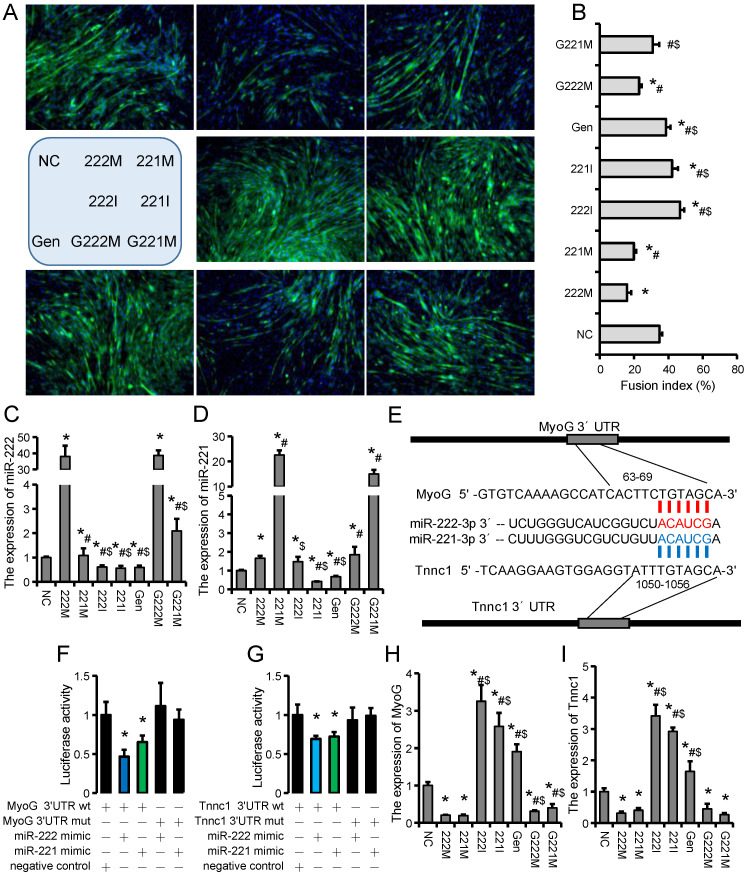
Effects of GEN and miR-221/222 family on C2C12 myoblast differentiation. (**A**) MyHC immunofluorescence staining of C2C12 myotubes after different treatments. (**B**) The fusion rate of C2C12 myotubes after different treatments. (**C**,**D**) Expression of miR-222 (**C**) and miR-221 (**D**) in C2C12 myotubes after different treatments. (**E**) The binding sites prediction of *MyoG*/*Tnnc1* and miR-221/222 family. (**F**,**G**) Fluorescence intensity of the dual-luciferase reporter assay system for the potential binding sites of miR-221/222 family and *MyoG* (**F**)**/***Tnnc1* (**G**). (**H**,**I**) Expression of *MyoG* (**H**) and *Tnnc1* (**I**) in C2C12 myotubes after different treatments. *n* = 3. * *p* < 0.05, compared with the NC group, # *p* < 0.05, compared with 222 M group, $ *p* < 0.05, compared with 221 M group.

**Figure 6:**
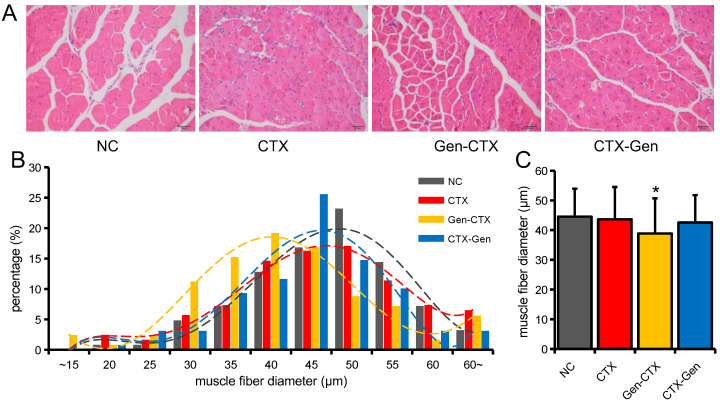
Effects of different treatment time of GEN on skeletal muscle regeneration. (**A**) HE staining of TA muscle of mice 21 days after injury. (**B**) Distribution frequency of TA muscle fiber diameter. (**C**) Mean muscle fiber diameter of TA muscle. *n* = 3. * *p* < 0.05, compared with the NC group.

Myotube differentiation and fusion are required for skeletal muscle regeneration. Compared with the NC group, the myotube fusion rate was significantly decreased in the 221 M and 222 M groups, while significantly increased in the Gen, 221 I, and 222 I groups (Figure 5A,B). Compared with the 221 M group, the myotube fusion rate was significantly increased after GEN treatment (Figure 5A,B). The myotube fusion rate was also significantly increased after GEN treatment compared with the 222 M group (Figure 5A,B). Similar to the proliferative stage, GEN also significantly inhibited the expression of miR-221 and miR-222 in C2C12 myotubes (Figure 5C,D). The biological functions of miRNAs depend on their target genes. Through target gene prediction and sequence alignment analysis, we found that *MyoG* and *Tnnc1* may be potential target genes of the miR-221/222 family (Figure 5E). The binding relationship of *MyoG* and *Tnnc1* to miR-221/222 was further confirmed by a dual-luciferase reporter system (Figure 5F,G). Compared with the NC group, 221 M and 222 M significantly inhibited the expression of *MyoG* and *Tnnc1*, while 221 I, 222 I and Gen significantly promoted the expression of *MyoG* and *Tnnc1* (Figure 5H,I). We further compared the effect of GEN treatment on skeletal muscle regeneration at different time points of muscle injury, and found that GEN treatment at different stages can accelerate the repair process, but GEN pretreatment before injury will cause regeneration muscle fiber diameter was significantly reduced (Figure 6A–C). These results suggest that the beneficial effects of GEN are mainly in the middle and late stages of skeletal muscle regeneration, and GEN improves skeletal muscle regeneration mainly by inhibiting the persistence of macrophage inflammation and promoting the differentiation and fusion of myoblasts. Premature GEN treatment inhibits the necessary proliferation of myoblasts and macrophages, which in turn leads to a reduction in the diameter of new muscle fibers.

## 3. Discussion

The regenerative capacity of skeletal muscle is of great significance for maintaining the homeostasis of skeletal muscle tissue and ensuring normal physiological functions under conditions of disease and injury [2]. In this study, we found that GEN might affect muscle repair after injury by regulating the expression of miR-221/222. As an important class of epigenetic regulators, microRNAs have been widely reported to be involved in the regulation of skeletal muscle development [24]. The six miRNAs found in this study with the opposite trend in ESR1 expression were all reported to be involved in the regulation of skeletal muscle development. miR-142a-5p is involved in mitophagy and apoptosis of atrophic muscles by targeting *MFN1* [25]. miR-15b-5p is involved in myotube differentiation by targeting *SETD3* [26]. Inhibition of miR-200c can promote myotube differentiation [27]. miR-223-3p promotes skeletal muscle regeneration by regulating inflammation [28]. miR-221/222 regulate skeletal muscle differentiation through the MAPK pathway [29]. These results suggest the importance of co-highly expressed miRNAs on skeletal muscle development 1 and 3 days after muscle injury.

GEN has been reported to affect skeletal muscle development by regulating protein degradation [30], immunity [31], oxidative stress [32], and myotube differentiation [22]. Notably, GEN has been reported to regulate the expression of miR-1260b [19], miR-223 [20], miR-155 [33], miR-20a [34], miR-451 [35], miR-221/222 [36], etc. Among them, miR-222 expression was inhibited by GEN in PC-3 cells [36], MCF-7 cells [37], and U87-MG cells [38], adipocytes [21], myoblasts [22]. Intriguingly, this regularity is not found in other miRNAs. In this study, GEN was also found to inhibit the expression of miR-221 and miR-222 in myoblasts and RAW264.7 cells. These results suggest that GEN has a broad inhibitory effect on miR-221/222. miR-221/222 have been reported to play a role in promoting cell proliferation in a variety of cells [39,40], and in this study, we also found that overexpression of miR-221 and miR-222 significantly promoted myoblasts and RAW264.7 cell proliferation. Both GEN treatment and inhibition of miR-221/222 significantly inhibited cell proliferation.

In addition to the activation, proliferation and differentiation of skeletal muscle cells, muscle injury and pathology can also induce a series of inflammatory responses at the injury site [41]. There are only a small number of macrophages in normal muscle tissue, and a large number of macrophages infiltrate the injured muscle tissue from 1 to 3 days after injury [42,43]. The main role of macrophages is to remove the cellular debris produced by muscle damage, thereby promoting the repair of muscle damage [41]. Skeletal muscle mainly expresses pro-inflammatory factors in the first 3 days after injury, which promotes the removal of cell debris at the injury site. After 3 days of injury, pro-inflammatory factors gradually decrease and anti-inflammatory factors gradually increase to avoid excessive tissue damage [12,41]. *IL-6* and *TNFα* are two major pro-inflammatory factors [44], and this study found that both GEN and inhibition of miR-221/222 could significantly inhibit the LPS-induced increase in the expression of *IL-6* and *TNFα* in RAW264.7 cells. At the same time, we found that GEN treatment before muscle injury resulted in a significant reduction in the diameter of new muscle fibers at 21 days, which may be caused by insufficient proliferation of muscle satellite cells and macrophages due to premature GEN treatment. In subsequent experiments, the relationship between GEN and inflammation during muscle regeneration can be further explored.

*MyoG* (Myogenin) is a marker gene for skeletal muscle differentiation [45], and *Tnnc1* (troponin C1) is an important regulator of striated muscle contraction [46]. In this study, we found and confirmed the binding relationship of miR-221/222 to *MyoG* and *Tnnc1*. In the late stage of muscle injury, the differentiation and fusion of myotubes were the main factors [11], and both GEN and inhibition of miR-221/222 significantly promoted the differentiation and fusion of myotubes. Meanwhile, GEN treatment and inhibition of miR-221/222 also significantly promoted the expression of *MyoG* and *Tnnc1* in myotubes. This study found that GEN treatment at 3 days after injury did not affect the diameter of new muscle fibers, probably because the adverse effects of GEN on cell proliferation were avoided at this time. In addition, because GEN promoted the differentiation and fusion of myotubes, the morphological structure of newborn muscle fibers in the CTX-Gen group 21 days after muscle injury was closer to that of uninjured muscle (the cross-section of muscle fibers was more regular, and the central nucleus muscle fibers began to decrease). These results suggest that the beneficial effects of GEN mainly accelerate the regeneration process by promoting the differentiation and fusion of myotubes in the middle and late stages of skeletal muscle regeneration.

## 4. Materials and Methods

### 4.1. Animals and Treatment

8-week-old female C57BL/6J mice were purchased from Chengdu Dashuo Experimental Animal Co, Ltd. (Chengdu, China). All mice were housed in a dedicated experimental animal room at 23 °C with a natural light cycle. During the experiment, all mice had free access to food and water. Animal care and all procedures were approved by the Animal Care and Ethics Committee of Sichuan Agricultural University.

### 4.2. Skeletal Muscle Injury Model

The construction of the muscle injury model refers to previous reports [23]. After one week of environmental acclimation, the mice were injected with cardiotoxin (CTX, 10 μM, Sigma-Aldrich, Inc., St. Louis, MO, USA) into the tibialis anterior (TA) muscle to construct a skeletal muscle injury model, 40 μL per mouse. Mice were given GEN (Gen, JINGZHU BIO-TECHNOLOGY (Nanjing, China), 10 mg/kg body weight per day) by gavage at 3 days before muscle injury (Gen-CTX) or 3 days after muscle injury (CTX-Gen), using olive oil as a solvent, 0.2 mL per mouse.

### 4.3. Skeletal Muscle Tissue Sections

The TA muscle of mice were taken and fixed in 4% paraformaldehyde for 24 h; then, the samples were dehydrated, embedded and sectioned, and finally stained with hematoxylin–eosin (HE). The diameter of muscle fibers was counted using Image-Pro Plus 6.0 software.

### 4.4. Cell Culture and Transfection

C2C12 myoblasts, RAW264.7 cells and HeLa cells (Stem Cell Bank of Chinese Academy of Sciences, Beijing, China) were cultured in a carbon dioxide incubator (37 °C, 5% CO_2_). C2C12 myoblasts and RAW264.7 cells were cultured in growth medium (GM), which was composed of 10% fetal bovine serum (FBS, Gibco, Jenks, OK, USA) and 90% DMEM (Gibco). C2C12 myotubes were differentiated from C2C12 myoblasts. When the density of C2C12 myoblasts reached about 80%, the differentiation medium (DM) was used for culture, which contained 2% horse serum (HS, Gibco) and 98% DMEM. Gen was dissolved in dimethyl sulfoxide (DMSO, Solarbio, Beijing, China), and lipopolysaccharide (LPS, Sigma, MO, USA) was dissolved in phosphate-buffered saline (PBS). Lipofectamine 3000 (Invitrogen, Guangzhou, China) was used to transfect miR-222 mimic (222 M, 50 nM), miR-221 mimic (221 M, 50 nM), miR-222 inhibitor (222 I, 50 nM), miR-221 inhibitor (221 I, 50 nM), Gen (10 μM ), LPS (10 μM) and negative control (NC, 50 nM, GenePharma, Shanghai, China). C2C12 myoblasts and RAW264.7 cells in the proliferation stage were collected 24 h after transfection. RAW264.7 cells were collected 12 h after LPS treatment. C2C12 myotubes were collected after 5 days of differentiation.

### 4.5. Real-Time Quantitative PCR

Total RNA was extracted from mouse tissues and cells with TRIzol reagent (TaKaRa, Dalian, China). Then, RT-qPCR was performed in a CFX96 real-time quantitative PCR detection system (Bio-Rad, Hercules, CA, USA) according to the instructions of SYBR Premix Ex Taq II (2×) kit (TaKaRa, Dalian, China). The relative expression levels of mRNAs and miRNAs were calculated using the 2^−∆∆Ct^ method. ACTB was selected as the internal reference for mRNA, and U6 was selected as the internal reference for miRNA. The primer sequences used in this study are shown in Table S1.

### 4.6. Dual Luciferase Reporting System

The psiCHECK™-2 vector was used in the dual-luciferase reporter system, as described in previous studies. The psiCHECK™-2 vector (containing the 3′ UTR fragment of *MyoG* and *Tnnc1*), miR-222 mimic and miR-221 mimic, negative control or GEN was co-transfected into HeLa cells using Lipofectamine 3000. Cell samples were collected 48 h after co-transfection. Firefly and Renilla fluorescence were measured using the Dual Glo Luciferase Assay System (Promega, Madison, WI, USA).

### 4.7. EdU Proliferation Assay and Myotube Immunofluorescence Staining

Cell proliferation was detected using the EdU reagent kit (Ribobio, Guangzhou, China). At a time of 24 h after transfection, 10 μM EdU was added and incubated for 2 h. Fixation and EdU staining were performed according to the instructions. C2C12 myotubes (after 5 days of differentiation) were washed with PBS and then fixed with 4% paraformaldehyde for 20 min. Cell membranes were permeabilized with 0.2% Triton X-100 (Solarbio) for 10 min at room temperature. Nonspecific antibody binding was blocked with 5% goat serum (Servicebio, Wuhan, China) for 1 h. MyHC antibody (Servicebio) was incubated at 4 °C for 12 h and finally incubated with a Fitc-labeled secondary antibody (Servicebio) for 2 h.

### 4.8. Statistical Analysis

All data were analyzed using the SPSS 20.0 software (IBM, Almond, NY, USA) and expressed as means ± standard deviation (SD). One-way analysis of variance (ANOVA) was then used to determine whether there were any significant differences between the means of two groups, with the significance threshold set at *p* ≤ 0.05.

## 5. Conclusions

In conclusion, in this study, we report that GEN acts as a natural regulator of miR-221/222 and their target genes *MyoG* and *Tnnc1* in the process of skeletal muscle regeneration. By analyzing the changes in muscle satellite cells in the process of skeletal muscle regeneration, combining with in vitro and in vivo experiments, we found that the optimal use time of GEN was in the middle and late stages of the regeneration process. GEN improves skeletal muscle regeneration mainly by inhibiting inflammation and promoting the differentiation of muscle satellite cells.

## Data Availability

The corresponding author can be contacted if necessary.

## Supplementary Materials

The following supporting information can be downloaded at: https://www.mdpi.com/article/10.3390/ijms232113482/s1
