# Supplementary material for: Genistein Promotes Skeletal Muscle Regeneration by Regulating miR-221/222
Source: Int J Mol Sci. 2022 Nov 3;23(21):13482. doi: 10.3390/ijms232113482 (PMC9654045; doi:10.3390/ijms232113482)
Supplement: Supplementary file 1 [file ijms-23-13482-s001.zip › ijms-1953790_FigureS1.pdf]

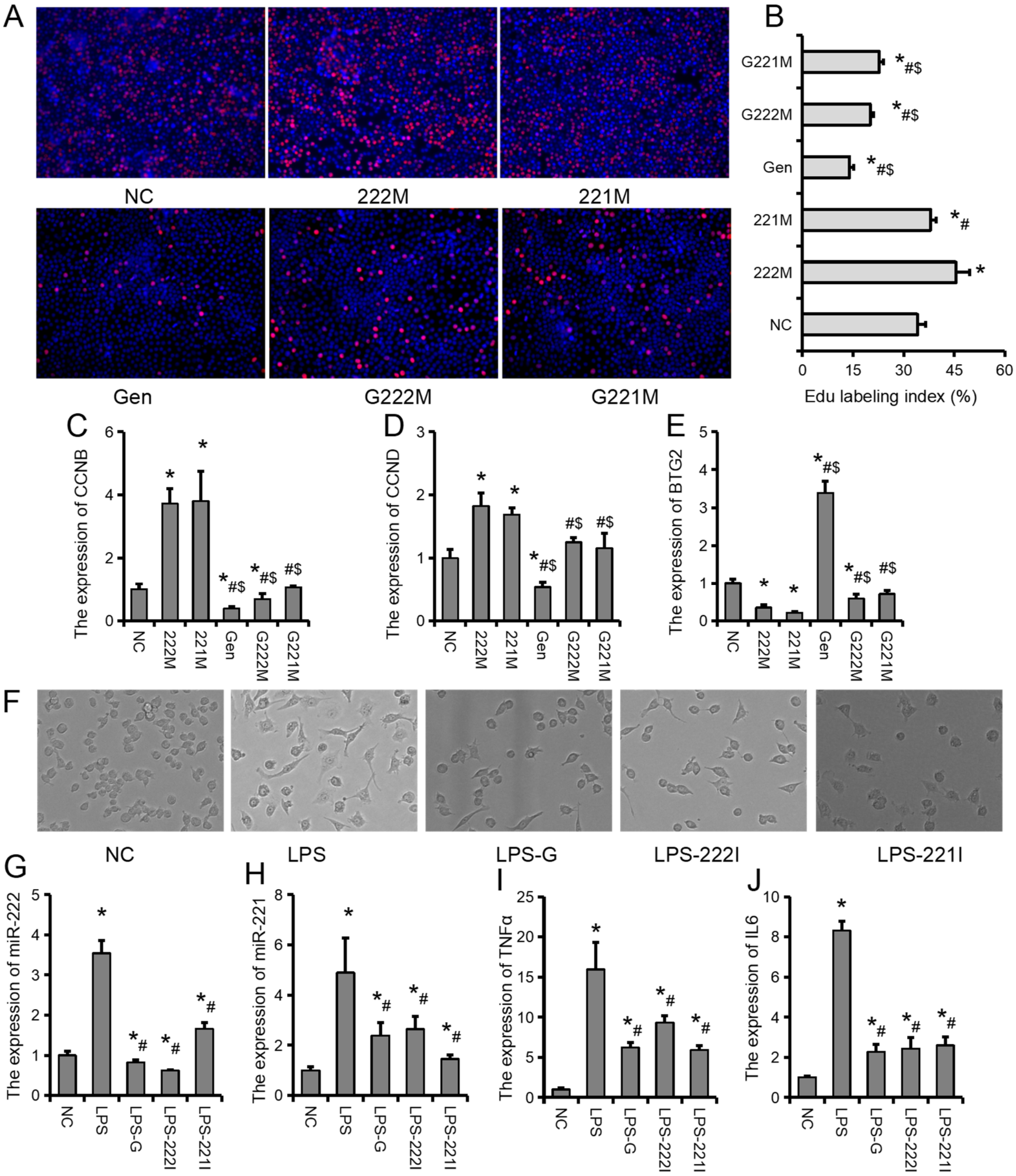

Figure S1. GEN and miR-221/miR-222 inhibited RAW264.7 cell proliferation and LPS-induced inflammatory factor expression. (**A**–**B**) EdU staining (**A**) and counting (**B**) of RAW264.7 cell after different treatments. (**C**–**E**) Expression of *CCNB* (**C**), *CCND* (**D**) and *BTG2* (**E**) in RAW264.7 cell after different treatments. (**F**) Morphological characteristics of RAW264.7 cells after different treatments. (**G**–**J**) Expression of miR-222 (**G**), miR-221 (H), *TNFα* (**I**) and *IL6* (**J**) in RAW264.7 cell after different treatments. **B**–**E**: * $p < 0.05$, compared with the NC group, # $p < 0.05$, compared with 222 M group, \$ $p < 0.05$, compared with 221 M group. **G**–**J**: * $p < 0.05$, compared with the NC group, # $p < 0.05$, compared with LPS group.
